# First report of the presence of *Necrodes littoralis* (L.) (Coleoptera: Silphidae) on a human corpse in Italy

**DOI:** 10.1111/1556-4029.14821

**Published:** 2021-08-30

**Authors:** Teresa Bonacci, Federica Mendicino, Francesco Carlomagno, Domenico Bonelli, Chiara Scapoli, Marco Pezzi

**Affiliations:** ^1^ Department of Biology, Ecology and Earth Sciences University of Calabria Arcavacata di Rende, Cosenza Italy; ^2^ Department of Life Sciences and Biotechnology University of Ferrara Ferrara Italy

**Keywords:** colonization, human, indoor, Italy, *Necrodes littoralis*, Silphidae, suburban area

## Abstract

The colonization of a human body by *Necrodes littoralis* (L.) (Coleoptera: Silphidae) is reported for the first time in Italy. This species is both necrophagous and predator of necrophagous fauna. The body colonized by the coleopteran was found indoors, in an advanced decomposition stage, in a suburban area of Cosenza (Calabria, Southern Italy) in November. Insects (adults, puparia and larvae) were collected on and around the body. Puparia and larvae were raised in the laboratory until the adult stage for morphological identification, which was carried out through taxonomical keys. Besides *N*. *littoralis*, also the presence of *Calliphora vicina* Robineau‐Desvoidy, *Chrysomya albiceps* (Wiedemann) (Diptera: Calliphoridae), *Hydrotaea dentipes* (Fabricius) (Diptera: Muscidae), and *Creophilus maxillosus* (L.) (Coleoptera: Staphylinidae) was detected. *Necrodes littoralis* is a species of forensic interest because it may colonize human and vertebrate corpses and has been reported elsewhere in Europe.

**Highlights**

- First report of colonization of a human corpse by the coleopteran *Necrodes littoralis* in Italy.
- The corpse was in advanced decomposition stage and found indoors in a suburban area.
- Three species of Diptera and another one of Coleoptera were also found on and nearby the body.

## 1 INTRODUCTION

Among insect orders, Coleoptera are relevant for forensic investigations, together with Diptera. Some species of this order directly feed on corpses but others predate necrophagous fauna, interfering with the colonization of remains. Forensic entomologists rely both on age of immature insects found on corpses and on the arthropod successional pattern associated with different decay stages [1, 2, 3, 4, 5]. *Necrodes littoralis* (L.) (Coleoptera: Silphidae) is of forensic interest because it colonizes and breeds on human and vertebrate remains. This necrophagous species is also a predator of dipteran larvae [6, 7]. Although common in Europe, it has never been reported in Italy as colonizer of human bodies. Here, we describe for the first time the presence and activity of *N*. *littoralis* on a well‐decomposed human corpse discovered indoors in an abandoned sports facility in a suburban area of Cosenza (Calabria, Southern Italy).

## 2 CASE DESCRIPTION

**FIGURE 1:**
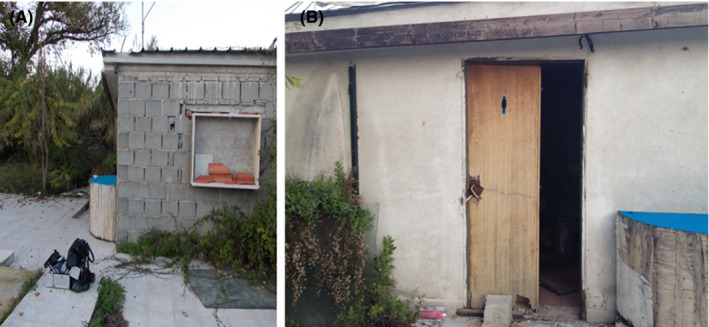
Abandoned sport facility in a suburban area near Cosenza (Calabria, Southern Italy) where the body of a 50‐year‐old man was found. (A) External view. (B) Open door for access to the building [Color figure can be viewed at wileyonlinelibrary.com]

**FIGURE 2:**
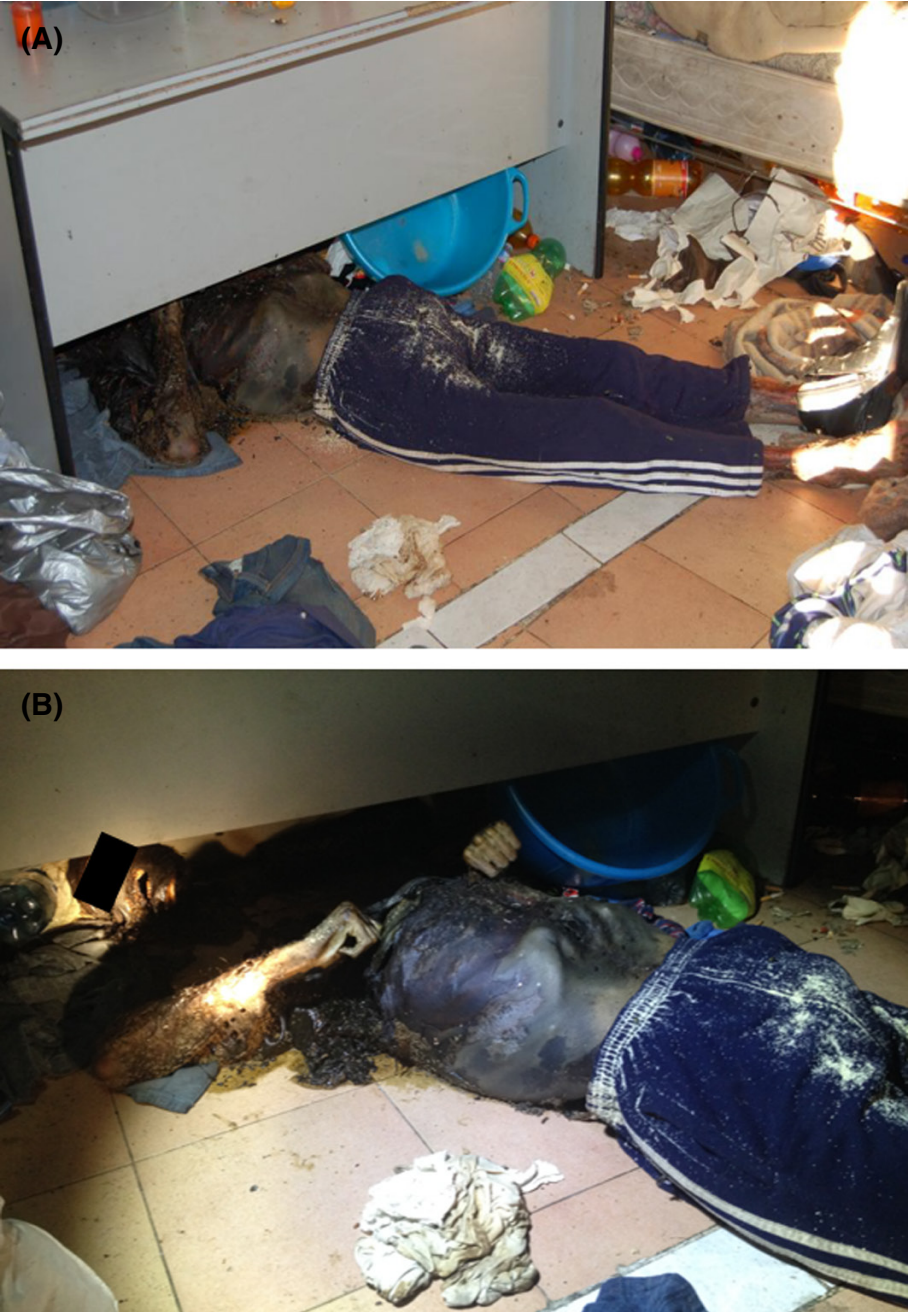
Human body found inside the facility. (A) Body on the floor, under a table, surrounded by garbage and dirty clothes. (B) Detail of the body in supine position showing the advanced stage of decomposition [Color figure can be viewed at wileyonlinelibrary.com]

A 50‐year‐old man was found dead in a suburban area of the city of Cosenza (Calabria, Southern Italy) on November 17, 2013 (39°18′55.83″N; 16°14′22.02″E), at 4.00 p.m. The corpse was found indoors in the dressing room of an abandoned and windowless sport facility, whose door, facing south and damaged, was half open (Figure 1). Outside the building, the vegetation was composed by *Ailanthus altissima* (Mill.) Swingle (Sapindales: Simaroubaceae), *Arundo donax* L. (Poales: Poaceae), and *Rubus ulmifolius* Schott (Rosales: Rosaceae). The corpse was discovered in an advanced decomposition stage, on the floor, in a supine position (Figure 2) and wearing only tracksuit trousers and a shoe on his left foot. In the room, paved with ceramic tiles, there was a cot with a mattress and a desk. A lot of garbage and dirty clothes surrounded the body. The autopsy, carried out 2 days after the discovery of the corpse, was not able to establish the cause of death. Moreover, since the body was discovered in an advanced decomposition stage, toxicological analyses were not possible. When the body was found, the indoor air temperature was 16.1°C and the outdoor temperature 15.6°C. The mean temperature recorded 10 days before the discovery of the body in Cosenza was 15.0 ± 2.1°C (https://www.cfd.calabria.it). On and near the corpse, dipteran larvae, puparia, and coleopteran adults were hand collected by entomological tweezers. The coleopteran adults were immediately stored in test tubes with 60% ethanol. A total of 193 dipteran larvae were collected: 68 of them were boiled for 90 s and preserved in plastic test tubes in 90% ethanol. The other dipteran larvae were reared in the laboratory in mesh‐covered plastic boxes containing 100 g of minced pig liver at 20°C, until development of puparia, which were then transferred to boxes with sand until emerging of adults. The puparia found on and around the body were separately placed in boxes with sand and kept at 20°C until emerging of adults.

The dipteran larvae and emerged adults were identified using taxonomical keys [8, 9]. The dipteran species detected were *Calliphora vicina* Robineau‐Desvoidy, *Chrysomya albiceps* (Wiedemann) (Diptera: Calliphoridae), and *Hydrotaea dentipes* (Fabricius) (Diptera: Muscidae). The coleopteran adults were identified using taxonomical keys [10, 11] and belonged to the species *Creophilus maxillosus* (L.) (Coleoptera: Staphylinidae) and *Necrodes littoralis* (L.) (Coleoptera: Silphidae). Based on evaluation of the larval stages of *C*. *vicina* found on the body, the death of the man was estimated between November 3 and 4, 2013.

## 3 DISCUSSION

This is the first report on the presence of *Necrodes littoralis* on a human body in Italy. The other dipteran species found on the body, *C*. *vicina* and *Ch*. *albiceps* and the coleopteran *Cr*. *maxillosus* have already been reported on human bodies in Italy, the first two indoors and outdoors, and the third only outdoors [12, 13, 14, 15, 16, 17, 18]. Concerning *H*. *dentipes*, this species was previously found on a human body in Northern Italy, see Grzywacz et al. [19], but it has been found for the first time in Southern Italy during this study. Among Coleoptera, the species belonging to the family Silphidae are widely distributed around the world. The family is divided into two subfamilies, Silphinae and Nicrophorinae [3], and includes about 210 species, 44 of which are present in Italy [20]. Silphidae are scavengers that contribute to recycling organic matter [21] and are able to consume dead vertebrates [22]. They are frequently found on human remains [7, 23, 24, 25] and on experimental animal models [26, 27, 28, 29]. Several forensic studies have reported their presence in insect successional patterns but little attention has been devoted to their role as forensic indicators in human cases [27, 30].

*Necrodes littoralis*, with a Palearctic distribution and widespread throughout Europe [31], has necrophagous and predatory habits [7] and more frequently colonizes large vertebrate carcasses [32]. It has been found in dry meadows [32], forest areas [26, 27, 28, 33, 34], and rural areas [27, 29, 35].

The species has been reported in Italy on animal carrions and in pitfall traps with rotting meat bait [36]. Concerning human bodies, many cases of colonization by *N*. *littoralis* have been reported outside Italy. Larvae and adults of this species were found in Spain in the soil near a body of a young man, and adults were found on the body during the autopsy [37]. In France, the species was found in 154 forensic cases out of 1028, examined from 1990 to 2013 [7]. According to this study, the species mainly reached the corpses during the advanced stages of decomposition and only rarely during the other stages. In Poland, *N*. *littoralis* was found on human bodies in 2015 [38] and 2017 [39]. Outside Europe, *N*. *littoralis* was found on a human corpse discovered in a forest area in South Korea [40]. The third instar larvae of this species found in association with empty puparia of *Phormia regina* (Meigen) (Diptera: Calliphoridae) were used for estimation of post‐mortem interval (PMI) of a human body discovered in an advanced decomposition stage in Western Poland [38].

In the reported case from Calabria, the species was found associated with the thermophilic species *Ch*. *albiceps* and *C*. *vicina*. The access to the building through the open door and the state of total neglect of the area where the corpse was found, as well as the late discovery of the body, may have favored the indoor colonization by *N*. *littoralis*.

The presence of *N*. *littoralis* is relevant from a forensic point of view because this coleopteran species may predate the larvae of necrophagous dipterans, thus interfering with a correct estimation of PMI. The first report of this species on a human body in the Italian territory and in an indoor suburban area is therefore important not only for ecological studies but also for forensic investigations.
